# The costs of being big in a warmer world

**DOI:** 10.1093/conphys/cox022

**Published:** 2017-03-24

**Authors:** Lisa M. Komoroske

**Affiliations:** 1 National Research Council under contract to Southwest Fisheries Science Center; Marine Mammal and Turtle Division, National Marine Fisheries Service, National Oceanic and Atmospheric Administration, 8901 La Jolla Shores Drive, La Jolla, CA 92037, USA

It takes more energy for fish to survive in warm waters. But, it turns out that the costs may be even higher for larger individuals compared to smaller ones, according to a new study by Messmer *et al.*(2016). If resources become limited for big fish at higher temperatures, climate change could dramatically impact fisheries productivity and ecosystem health.

Scientists first documented that animals have smaller body sizes in warmer environments back in the 19th century. Today, scientists know from laboratory studies that most ectotherms (i.e. previously called cold-blooded animals), such as fish, grow faster but to smaller maximum body sizes at higher temperatures. Yet, the mechanisms driving these patterns are somehow still unknown. Hypotheses have been proposed to explain these patterns. And, theoretical models have been built, some of which project that ocean warming will result in an alarming 14–24% decrease in maximum body mass of marine fishes globally by the year 2050! Yet many of these models have been criticized because they do not have a solid physiological basis. So, discovering the physiological drivers of size–temperature relationships has an especially important applied context—forecasting climate change impacts on global food supply and ecological sustainability.

Taking a first bite out of this problem, Messmer *et al.*(2016) set out to test one of the hypotheses: *increased metabolic requirements limit large body sizes under warmer conditions.* To figure this out, the team collected coral trout, a reef fish highly prized among fishers across the Indo-Pacific and an icon of the Great Barrier Reef. The team held the fish in the laboratory at either current or future projected summer temperatures. Then they measured how much oxygen the different sized coral trout consumed while either resting or exercising to understand how the fish's metabolism responded to warmer conditions.

At temperatures projected for the future with climate change, fish needed more oxygen to fuel their metabolism, agreeing with many past studies showing that metabolism increases with temperature. But the team also discovered that bigger fish needed even more energy in warmer waters than the smaller individuals. For example, maximum metabolic rates increased ~35-fold from small to big fish at the lower (current day) temperatures but by a staggering 44-fold for fish held at the higher (near future) temperatures! Messmer's team also found that smaller fish had a greater tolerance for high temperatures than larger fish. Together, these results indicate that bigger coral trout are more thermally sensitive and could be disproportionately impacted by ocean warming.

So is the mechanism underpinning temperature and body size correlations related to heightened metabolic demands? Messmer *et al*. (2016) provide empirical evidence that supports this hypothesis and will hopefully motivate future studies to dive deeper into the many remaining questions: how do these relationships change with development? How do species differ? And, *‘How big is too big?’* in terms of metabolic demands?

Indeed, there are many more questions to answer to solve this puzzle that has been eluding scientists for over a century. One thing is clear, however. Conservation physiologists need to work with fisheries scientists to connect the fundamental mechanisms and real-world implications of future, warmer oceans on global fisheries and marine ecosystems.

**Figure:**
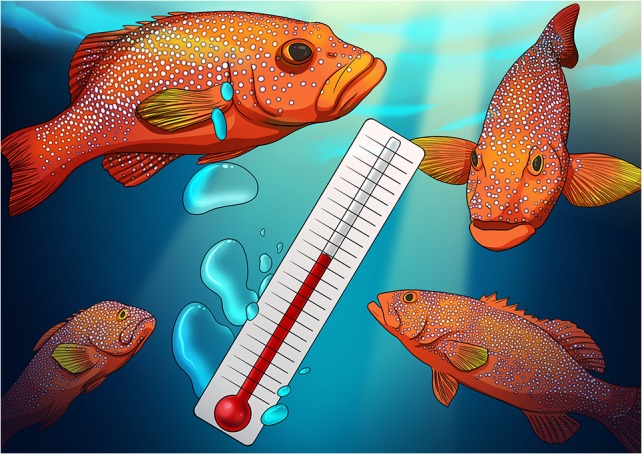

Illustration by Erin Walsh; Email: ewalsh.sci@gmail.com
